# Congo Red Unmasks the Culprit Behind Gastrointestinal Bleeding

**DOI:** 10.7759/cureus.111131

**Published:** 2026-06-19

**Authors:** Claire C Russell, Khaleel Quasem, Caleb M Glover, Edward Cay, Dorian Jones

**Affiliations:** 1 Internal Medicine, Michigan State University, Lansing, USA; 2 Internal Medicine, McLaren Greater Lansing, Lansing, USA; 3 Gastroenterology, McLaren Greater Lansing, Lansing, USA

**Keywords:** al amyloid, gastrointestinal pathology, gi amyloidosis, obscure gastrointestinal bleeding, oral chemotherapy

## Abstract

Immunoglobulin light chain (AL) amyloidosis arises from a plasma cell dyscrasia and is characterized by extracellular deposition of misfolded light chains. Gastrointestinal (GI) involvement is uncommon but clinically important because amyloid-related mucosal ischemia and vascular fragility can precipitate significant bleeding.

A 65-year-old woman with systemic AL (lambda) amyloidosis with cardiac and renal involvement and stage II IgA lambda multiple myeloma presented with several hours of acute hematochezia. Her hemoglobin was 9.6 g/dL on presentation. Computed tomography angiography showed no active contrast extravasation, with mild-to-moderate distal rectal wall thickening and increased intraluminal fluid in the left colon. Colonoscopy on hospital day three demonstrated an adherent sigmoid clot (22-40 cm from the anal verge) without active bleeding; the examination was limited by clot burden. Four days later, she developed recurrent large-volume hematochezia with hemoglobin nadir of 7.9 g/dL. Repeat colonoscopy on hospital day seven demonstrated sigmoid ulceration at the prior clot site, and biopsies were obtained. Routine histopathology showed ulcerated granulation tissue consistent with ischemic-type mucosal injury. Congo red staining was focally positive for amyloid deposition, confirming colonic amyloid involvement. She was discharged without recurrent hematochezia. This case underscores the diagnostic value of requesting amyloid evaluation (including Congo red staining) when endoscopic findings and routine histology are non-specific, particularly in patients with known or suspected plasma cell dyscrasia and recurrent or unexplained GI bleeding.

## Introduction

Biopsy-proven gastrointestinal (GI) involvement in primary systemic (AL) amyloidosis is uncommon, making severe, recurrent hematochezia from colonic amyloid clinically important and diagnostically non-routine [1]. Among patients with biopsy-proven GI amyloidosis, GI bleeding is a frequent presenting symptom; this case adds a notable pattern of delayed, large-volume rebleeding after an initial colonoscopy demonstrating an adherent clot without active hemorrhage [2]. Endoscopic abnormalities in luminal AL amyloidosis often include ulcerations and submucosal hematomas, and this case is notable for evolution from an “adherent clot” to a biopsy-proven sigmoid ulcer at the same site on repeat colonoscopy [3]. The recurrent hematochezia and apparent mucosal/vascular fragility in this presentation are mechanistically consistent with amyloid angiopathy-associated vessel fragility and impaired vasoconstriction, supporting amyloid as a plausible etiology when bleeding is recurrent or difficult to localize [4]. Because lower GI hemorrhage as an acute presentation of AL amyloid is uncommon, this case reinforces the literature-supported need for endoscopic biopsy (and repeat endoscopy when bleeding recurs) rather than anchoring on non-specific endoscopic findings [5]. Histologic confirmation remains pivotal in GI amyloidosis; here, the initial routine pathology suggested non-specific mucosal injury, with diagnosis established only after Congo red evaluation, supporting early, explicit requests for amyloid staining in high-risk patients such as those with plasma cell dyscrasia or known AL disease [6]. Finally, because diagnostic detection can be missed when the amyloid burden is small, the turning point in this case, biopsy-proven GI amyloidosis, aligns with evidence that enhanced evaluation can provide definitive diagnoses that may have been previously missed [7].

## Case presentation

Patient information

A mid-60s woman with stage IV systemic AL (lambda) amyloidosis with cardiac and renal involvement and stage II IgA lambda multiple myeloma on chemotherapy with cyclophosphamide, bortezomib, and dexamethasone (CyBorD) and venetoclax, anemia of chronic disease, thrombocytopenia, chronic kidney disease, hypertension, and hyperlipidemia presented with acute hematochezia.

Clinical findings

The patient had no prior history of gastrointestinal bleeding and was not receiving anticoagulation. She reported bowel incontinence with the first episode and approximately five episodes of hematochezia prior to emergency department arrival, with continued bleeding overnight. Family history included maternal Alzheimer’s disease and paternal diabetes mellitus.

On presentation, she was in no acute distress. Vital signs were stable - temperature 36.7°C, heart rate 86 beats per min, respiratory rate 14 breaths per min, and blood pressure 130/87 mmHg, with oxygen saturation 93% on room air. Abdominal examination demonstrated a soft, non-tender, non-distended abdomen without guarding or rigidity. Bright red blood was noted in her incontinence garment with visible active rectal bleeding. Extremities demonstrated intact pedal pulses with trace bilateral lower-extremity edema; neurologic examination was non-focal.

Timeline (hospital course summary)

Hospital day 0: presentation with acute hematochezia; initial labs notable for anemia and thrombocytopenia; CTA without active extravasation. Hospital day 0 to one: platelet transfusions administered; rapid bowel preparation completed. Hospital day three: colonoscopy showed adherent sigmoid clot (22-40 cm), no active bleeding; exam limited by clot burden. Hospital day seven: recurrent large-volume hematochezia; repeat colonoscopy showed sigmoid ulceration at the prior clot site; biopsies obtained. Discharge: no further hematochezia; outpatient follow-up arranged.

Diagnostic assessment

Initial laboratory evaluation showed anemia with hemoglobin of 9.6 g/dL (reported baseline: ~10.9 g/dL) with downtrend to a nadir of 7.9 g/dL during hospitalization in the setting of recurrent hematochezia. She also had severe thrombocytopenia requiring platelet transfusions (1 unit on presentation and a second unit on hospital day one). No laboratory evidence of coagulopathy was reported, and she was not receiving anticoagulation therapy.

Computed tomography angiography of the abdomen and pelvis revealed no arterial occlusion or active contrast extravasation. Imaging demonstrated increased intraluminal fluid within the left colon (possibly diarrhea or intraluminal blood products) and mild-to-moderate circumferential thickening of the distal rectum without a discrete mass; correlation with endoscopy was recommended.

Colonoscopy on hospital day three demonstrated an adherent clot within the sigmoid colon (22-40 cm from the anal verge) without evidence of active bleeding; the examination was limited due to clot burden. After recurrent large-volume hematochezia, repeat colonoscopy on hospital day seven demonstrated sigmoid ulceration and surrounding erythema at approximately 45 cm from the anal verge, and biopsies were obtained with cold forceps. Retroflexion revealed hypertrophied anal papillae.

**Figure 1:**
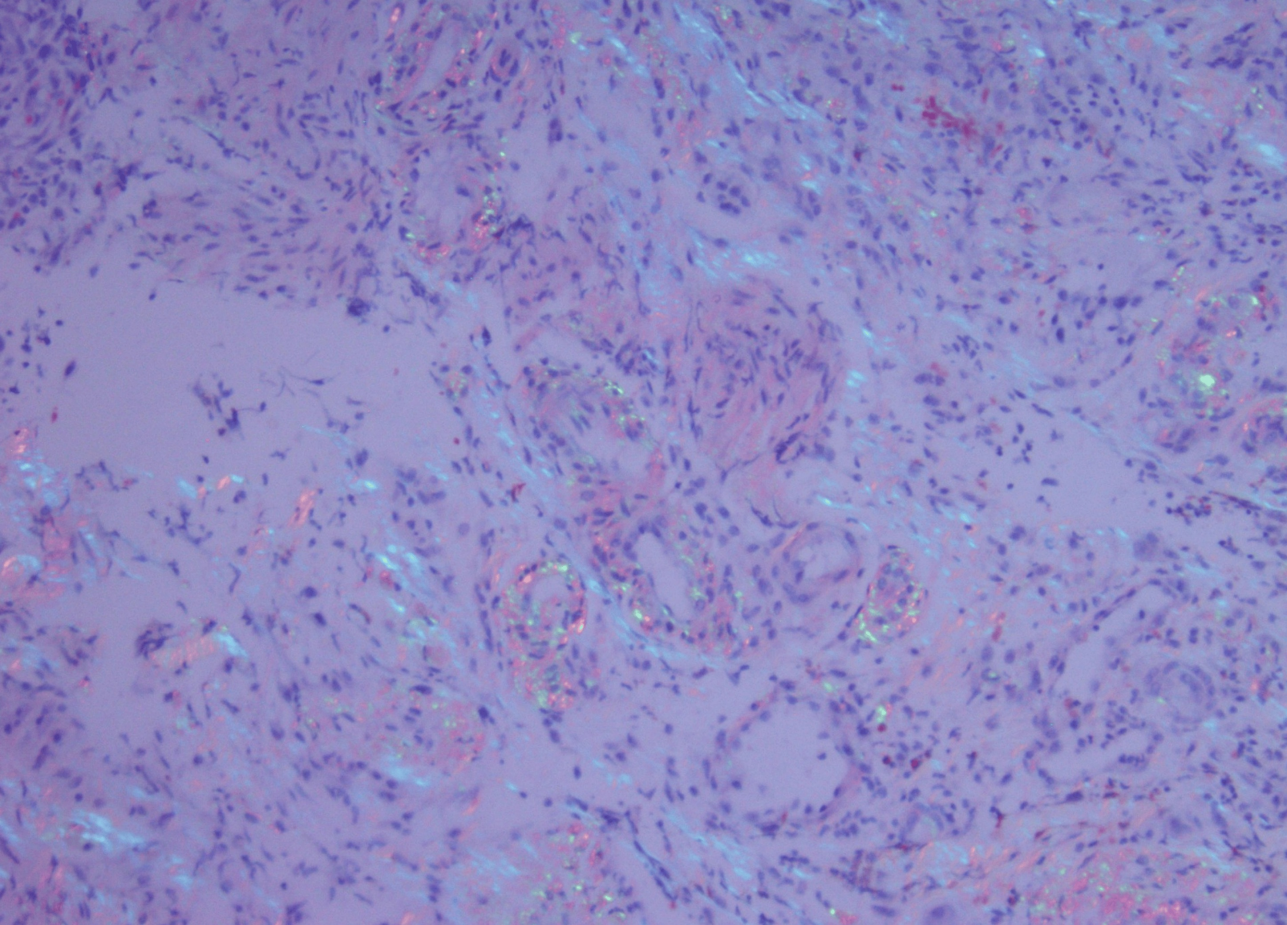
Apple-green birefringence on Congo red staining, confirming amyloid deposition.

Routine histopathologic evaluation of sigmoid colon biopsies demonstrated fragments of colonic mucosa with ulcerated granulation tissue consistent with ischemic-type mucosal injury, without evidence of chronic inflammatory changes, granulomas, dysplasia, or malignancy. Given her known systemic AL amyloidosis and recurrent bleeding without an alternative source, Congo red staining was performed and was focally positive for amyloid deposition with appropriate controls, confirming colonic amyloid involvement (Figure 1).

Flow cytometry, immunophenotyping, and molecular/genetic testing were not performed as part of the GI diagnostic evaluation because she had a pre-existing diagnosis of systemic AL amyloidosis and IgA lambda multiple myeloma established prior to presentation. The differential diagnosis included ischemic colitis, infectious colitis, medication-related mucosal injury, diverticular bleeding, and anorectal sources of bleeding.

Therapeutic intervention

Management focused on stabilization and supportive care for acute lower GI bleeding with close hemodynamic monitoring and serial complete blood counts. Platelet transfusions were administered for severe thrombocytopenia in the setting of active bleeding risk. Because CTA did not identify an active vascular source, endoscopic evaluation was pursued after rapid bowel preparation. No endoscopic hemostatic therapy was performed during the initial colonoscopy because active hemorrhage was not visualized, and the procedure was limited by clot burden. Surgical consultation was discussed as a contingency if bleeding recurred.

Following recurrent bleeding, repeat colonoscopy was performed with biopsy of the ulcerated sigmoid segment. Venetoclax was held for approximately one week due to concern for potential medication contribution and was restarted after hematology assessed that the bleeding pattern was more consistent with amyloid-related ulceration than a drug effect. Informed consent was obtained prior to endoscopic procedures after discussion of risks, benefits, and alternatives, and management reflected multidisciplinary input from gastroenterology and hematology/oncology, with consideration of surgical involvement if rebleeding occurred.

Follow-up and outcomes

Following discharge, the patient experienced prompt clinical stabilization with complete resolution of hematochezia and no further episodes of gastrointestinal bleeding. She did not require repeat endoscopic evaluation or surgical intervention for recurrent hemorrhage. She continued disease-directed therapy and reported adherence to chemotherapy (daratumumab, venetoclax, bortezomib, and dexamethasone) without missed doses. She continued symptom-focused pain management through palliative care. She remains under nephrology care and undergoes maintenance hemodialysis four times weekly while undergoing evaluation for renal transplantation. The index gastrointestinal hemorrhage occurred approximately 13 months prior to follow-up, without recurrent gastrointestinal bleeding.

Patient perspective

The patient reported no ongoing concerns related to her prior gastrointestinal hemorrhage and expressed relief that the bleeding resolved. She shared that she was grateful further endoscopic evaluation was not needed to confirm colonic amyloid involvement. She emphasized that, given the complexity of her systemic amyloidosis, multiple myeloma, and renal disease, she is focusing her energy on ongoing treatment, symptom control, and spending time with family.

## Discussion

Biopsy-proven gastrointestinal involvement in primary systemic (AL) amyloidosis is uncommon, which makes lower-GI bleeding from colonic deposits a diagnostically non-routine presentation [1]. In referral cohorts of GI amyloidosis, gastrointestinal bleeding is among the most frequent presenting symptoms, underscoring that hemorrhage can be a dominant clinical signal even when other GI complaints are absent [2]. Because AL amyloid only rarely presents as acute gastrointestinal hemorrhage, clinicians may initially anchor on more common etiologies of hematochezia unless systemic amyloid is already known or suspected [5]. Our patient’s recurrent, large-volume hematochezia illustrates how amyloid-related bleeding can declare itself abruptly despite stable hemodynamics and an initially non-diagnostic evaluation [2]. The case adds novelty by coupling established systemic AL disease with a focal colonic source that evolved over days, highlighting that symptomatic GI involvement may emerge even when overall GI amyloid prevalence is low [1]. In practice, this supports maintaining amyloidosis on the differential for unexplained lower-GI bleeding, particularly in patients with plasma cell dyscrasias or confirmed AL disease [5]. This learning point is reinforced by the literature showing that GI bleeding is common among patients with biopsy-proven GI amyloid, so missing the diagnosis can delay definitive attribution and targeted histologic testing [2].

Endoscopic abnormalities are found in most patients with luminal AL amyloidosis, including ulcerations and submucosal hematomas, but these patterns are not pathognomonic for amyloid and can mimic other colitides [3]. Our case was initially characterized by an adherent sigmoid clot without active bleeding, with subsequent rebleeding and evolution to a discrete ulcer in the same segment, illustrating how dynamic endoscopic appearances can be in GI amyloidosis [3]. When routine histology suggested only ischemic-type mucosal injury, the case highlighted that amyloid can be overlooked unless specifically evaluated on biopsy [7]. Definitive attribution required Congo red staining with polarized-light confirmation, reflecting the diagnostic value of apple‑green birefringence for amyloid detection [6]. This sequence supports the following key learning point: in high-risk patients with plasma cell dyscrasia and unexplained bleeding, requesting early amyloid staining can prevent diagnostic delay [6]. Published diagnostic evaluations show that enhanced methods can confirm small amyloid burdens or provide a definitive diagnosis that routine assessment would otherwise miss, thereby justifying repeat biopsy when suspicion remains high [7]. Accordingly, the novelty of this case lies in how a non-diagnostic initial pathology report became a definitive etiologic diagnosis only after targeted Congo red evaluation, emphasizing a process improvement opportunity in GI bleed workflows [7].

Amyloid angiopathy is thought to increase vessel fragility and blunt vasoconstrictive responses, creating a physiologic substrate for hemorrhage in systemic amyloidosis [4]. This mechanism helps explain why bleeding can be substantial even when imaging or endoscopy fails to capture active extravasation at a single time point [4]. In our patient, delayed large-volume rebleeding after an initially non-bleeding adherent clot is consistent with a lesion prone to recurrence as vascular integrity fluctuates [4]. Recognizing this pathophysiology reframes the endoscopic appearance from “mucosal injury” to amyloid-driven vascular vulnerability and supports prioritizing tissue-based confirmation when suspicion remains high [4]. It also underscores why supportive management, including close monitoring, transfusion as indicated, and multidisciplinary planning, is central when bleeding risk is amplified by systemic factors such as thrombocytopenia and renal failure [4]. From a learning standpoint, our patient’s prolonged bleeding-free follow-up after diagnosis suggests that achieving etiologic clarity can better align subsequent care with anticipatory risk reduction rather than repeated, non-directed investigations [4]. Overall, the case emphasizes that amyloid-related hemorrhage should be approached as a vascular fragility problem first and a localized “spot bleed” second, which informs counseling about recurrence and escalation pathways if rebleeding occurs [4].

Strengths

This case report is strengthened by a definitive tissue-based diagnosis with an instructive diagnostic pivot as follows: colonic amyloid involvement was confirmed on biopsy by Congo red staining after routine histopathology was initially non-specific, thereby strengthening etiologic attribution of the hemorrhage to amyloid-related mucosal and vascular fragility and underscoring a practical diagnostic step in high-risk patients. In addition, the clinical course provides a clear depiction of a clinically relevant diagnostic challenge with reproducible learning points, as an initial colonoscopy demonstrating an adherent clot without active bleeding was followed by delayed large-volume rebleeding and repeat colonoscopy identifying ulceration in the same segment, illustrating how gastrointestinal amyloidosis may present with evolving, non-specific endoscopic findings and supporting a low threshold for repeat endoscopy and targeted staining when bleeding recurs. The report also offers meaningful longitudinal outcome data consistent with CAse REport Guidelines (CARE) follow-up expectations, including extended follow-up from the index bleed over a 13-month period with absence of recurrent gastrointestinal hemorrhage, continuation of disease-directed therapy, ongoing renal replacement therapy, and active transplant evaluation, thereby strengthening the outcomes narrative beyond the inpatient setting. Finally, the manuscript benefits from multidisciplinary, patient-centered framing through integration of hematology/oncology, gastroenterology, nephrology, and palliative care involvement, alongside a patient perspective emphasizing symptom resolution, avoidance of further invasive testing, and prioritization of quality-of-life goals and family time amid a complex systemic disease burden.

Limitations

This report has several limitations inherent to case-based evidence. As a single-patient observation, it is not designed to establish incidence, quantify recurrence risk, or compare the effectiveness of diagnostic and therapeutic strategies for amyloid-associated lower gastrointestinal bleeding; therefore, it has limited generalizability and causal certainty. In addition, although the patient had established systemic AL (lambda) amyloidosis and the colonic biopsy was Congo red-positive, lesion-level amyloid subtyping at the gastrointestinal site (e.g., immunohistochemistry or mass spectrometry) was not documented, which would have further strengthened tissue-level confirmation and exclusion of alternative amyloid types. The clinical interpretation is also subject to residual confounding, as severe thrombocytopenia, advanced kidney disease/uremia, and concurrent antimyeloma therapy may each have contributed to bleeding susceptibility; while colonic amyloid deposition provides a unifying explanation for the focal lesion and recurrent hemorrhage, the relative contribution of these overlapping risk factors cannot be precisely quantified. Finally, follow-up outcomes are largely based on clinical course and patient report rather than standardized objective measures, and the manuscript would be strengthened by inclusion of structured post-discharge data (e.g., interval hemoglobin trends, transfusion requirements, and/or standardized symptom or quality-of-life assessments) and clear documentation regarding whether interval endoscopic reassessment was indicated or pursued.

## Conclusions

This case highlights colonic AL amyloid as an uncommon but high-stakes cause of recurrent hematochezia, including delayed large-volume rebleeding after an initially non-specific colonoscopic finding. Diagnosis required targeted histologic evaluation, emphasizing that Congo red staining should be explicitly requested when biopsy results are non-diagnostic and clinical suspicion persists in patients with known or suspected plasma cell dyscrasia. Early recognition of amyloid-associated vascular and mucosal fragility can prevent missed diagnoses, guide expectations for recurrence, and support coordinated multidisciplinary management focused on bleeding control and continuation of disease-directed therapy.
